# Mortality differences between immigrant and native children and youths in Denmark: a nationwide cohort study focusing on refugees and family-reunified immigrants

**DOI:** 10.1007/s00431-026-07159-z

**Published:** 2026-06-13

**Authors:** Yixin Wang, Ramune Jacobsen, Jørgen Holm Petersen, Marie Nørredam

**Affiliations:** 1 Department of Social and Clinical Pharmacy, University of Copenhagen, Copenhagen, Denmark; 2 Department of Pharmacy, University of Copenhagen, Copenhagen, Denmark; 3 Section of Biostatistics, Department of Public Health, University of Copenhagen, Copenhagen, Denmark; 4 Section of Health Services Research, Department of Public Health, University of Copenhagen, Copenhagen, Denmark; 5 Danish Research Centre for Migration, Ethnicity and Health, Department of Public Health, University of Copenhagen, Copenhagen, Denmark

**Keywords:** Immigrants, Refugees, Mortality, Minors, Adolescents, Children

## Abstract

Immigrant children and youths represent a growing population in Europe, yet studies on their health are scarce. Mortality is an indicator of both morbidity and healthcare access. This national register-based study aimed to compare all-cause and cause-specific mortality between natives and immigrants under 25 in Denmark. Using the Danish Migrant Cohort, we followed 82,461 immigrant and 473,244 native children and youths from 1993 to 2015 and from 2020 to 2022. Incidence rate (IR) was used to describe mortality by sex and age (in both groups) and by region of origin, migrant status, and length of stay (among immigrants). We used sex-stratified, age-adjusted Cox proportional hazard ratios (HR) to compare all-cause and cause-specific mortality between natives and immigrants overall and by region of origin and migrant status. We identified 156 deaths (IR = 3.20 per 10,000 person-years) and 815 deaths (IR = 2.49 per 10,000 person-years) among immigrant and native children and youths, respectively. Immigrants had higher overall mortality risk (HR 1.27, 95% CI 1.07–1.51). Group analyses showed elevated mortality in (i) males originating from Sub-Saharan Africa, (ii) male asylum seekers, and (iii) quota refugees of both sexes. Cause-specific mortality analyses showed higher suicide mortality in females from North Africa and Middle East and higher neurological disorder mortality in female quota refugees. With longer residence, mortality rates among immigrants decreased.

*Conclusions*: Immigrant children and youths in Denmark face excess mortality risks that diminish with longer stay. More research is needed to understand these differences and to reduce mortality among young immigrants.

**Table:** 

**What is Known:** • *Adult immigrants often show an overall mortality advantage compared with natives, but evidence on mortality for immigrant children and youths is limited and inconsistent.*
**What is New:** • *This nationwide Danish register–based cohort study shows that immigrant children and youths have higher all-cause mortality than natives.*

**Supplementary Information:**

The online version contains supplementary material available at 10.1007/s00431-026-07159-z.

## Introduction

By the end of 2024, 304 million people worldwide lived outside their country of birth. Over 51 million of whom—double the number from one decade prior—were forcibly displaced owing to persecution, war, or human rights violations [1]. Europe has become the region with the third-highest proportion of migrants: by 2024, it hosted 16% of all forcibly displaced people [2], and about 13% of its population were migrants [1]. Statistics Denmark defines immigrants as persons born abroad whose parents are also foreign-born and are not Danish citizens. By the end of 2023, there were more than 720,000 immigrants in Denmark, accounting for about 12% of the total Danish population [3]. Forcibly displaced immigrants mainly arrived as refugees and family-reunified immigrants and comprised 12.5% and 14.5% (respectively) of the total immigrant population in Denmark [4]. For clarity, the word “immigrant” is used when the main body of description is refugees and family-reunified immigrants in the following text, and the word “migrant” is used to refer to all types of immigrants.

Children are disproportionately affected by forced displacement. Worldwide, 40% of forcibly migrated people are children under 18, although children only account for 29% of the world’s population [5]. Europe holds 9 million forcibly displaced children [6]. In Denmark, similarly to the rest of Europe, about one-third of asylum seekers are children [7, 8].

Immigrants who were forced to migrate usually experience combined pre-, peri-, and post-migration risk factors [9, 10]. Many of them come from crisis-affected regions where adverse living conditions and traumatic experiences are common. Migration routes for refugees can be dangerous, presenting additional disease hazards. After arrival, immigrants commonly encounter the challenges of integration, which could lead to inadequate healthcare access. Immigrant children and youths may carry even higher physical and mental disease risks due to developmental vulnerability, making them more susceptible to malnutrition, poorer physical health, and mental disorders [11].

Mortality is a fundamental indicator of population health, as it reflects the combined effect of morbidity and access to healthcare. Several studies among the adult population [12–15], including one in Denmark [15], surprisingly found lower mortality in migrants compared to natives. This is known as the migrant mortality advantage (MMA). However, mortality studies among those who migrated as children showed elevated all-cause [16–18] and inconsistent cause-specific mortality risks [14, 16, 19, 20]. To our knowledge, no studies in Denmark to date have examined mortality of immigrant children and youths.

This study therefore compares the all-cause and cause-specific mortality of immigrant and native children and youths under 25 in Denmark. Furthermore, we aimed to investigate whether all-cause and cause-specific mortality risks in children and youths who migrated to Denmark varied by their length of stay, region of origin and migrant status.

## Methods

### Study setting

The study population was extracted from the Danish Migrant Cohort, obtained through the Aliens Register at the Statistical Department of the Danish Immigration Service. The main body of data was the cohort consisted exclusively of all refugees and family-reunified immigrants receiving their residence permit between 1 Jan 1993 and 31 Dec 2015, where a 6:1 frequency matching on sex and age with native Danes was performed. On the basis of this cohort, data from a smaller cohort constructed to study the impact of war on Ukrainian migration were pooled to construct the study cohort. The latter cohort contained all migrants (including work and education migrants) receiving residence permits between 1 Jan 2020 and 31 Dec 2022, where a 4:1 frequency matching on sex and age with native Danes was performed. Native Danes matched to immigrants were individuals born in Denmark with native Danish parents to avoid including descendants in both cohorts.

**Fig. 1:**
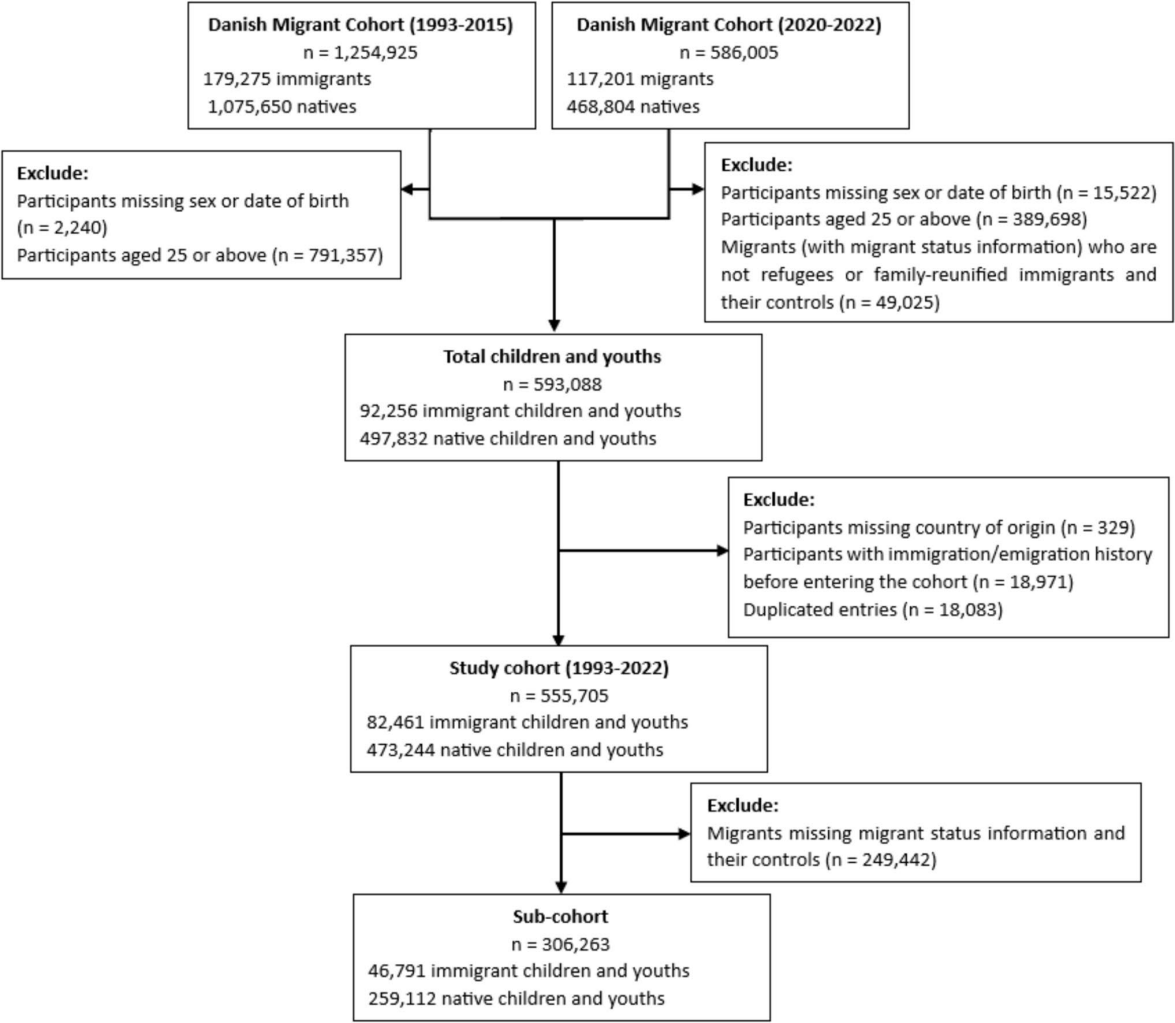
Flow chart of the process of study cohort construction

The target population was refugees and family-reunified immigrants under 25 based on the definition of “child” and “youth” by the World Health Organization (WHO) [21]. Migrants with other types of migrant status in the second cohort were excluded, along with their controls. This study applied complete case analysis. Individuals with missing information on sex, date of birth, country of origin, or who had migration history before entering the cohort were excluded. Missingness of data could be more prevalent in some groups due to the nature of Danish registers, but is not suspected to be related to individual outcomes. If an individual entered multiple times, only the first entry was included (Fig. 1). It should be noted that due to the randomness of re-entry, the study cohort was not strictly matched after excluding duplicate entries.

To analyse the association between migrant status and mortality, a sub-cohort was constructed by excluding migrants without registered migrant status. Information on migrant status was obtained from the Residence Permit Register established in 1997 [22], which was available for the Danish Migrant Cohort until 2021. Hence, immigrants who obtained residence permits between 1993 and 1997 or during 2022 and their controls were excluded from the sub-cohort.

### Data sources and variables

The study population was linked to mortality and sociodemographic data using the unique personal identification numbers assigned to all residents in Denmark. Data were retrieved from different national registers.

Information on date of birth, sex, country of origin, date of death, and immigration and emigration dates was extracted from the Danish Civil Registration System [23]. For immigrant children and youths, the cohort entry date was defined as the first immigration date. For native children and youths, the cohort entry date was defined as 1 Jan of the year when corresponding immigrants entered the cohort. Age at entry was calculated by extracting date of birth from the cohort entry date, then categorising into five groups: age 0–4, 5–9, 10–14, 15–19, and 20–24. Length of stay was calculated by extracting the cohort entry date from the date of the end of follow-up, then categorising into four groups: 0–4, 5–9, 10–14, and ≥ 15 years. Follow-up ended on date of death, first emigration date, date of 25th birthday, or end of study, whichever came first. Due to low case counts, all immigrant children and youths who stayed for 15 years or longer were grouped into one category. 5-year cutoffs were chosen as they are commonly used in epidemiology studies to balance between sparsity of cases and coarseness [24, 25]. The origin of immigrant children and youths was classified using the Global Burden of Disease super regions [26] and grouped into four categories: (1) Central Europe, Eastern Europe, and Central Asia; (2) North Africa and Middle East; (3) Sub-Saharan Africa; and (4) Other Regions, collecting remaining regions with low case counts.

For the sub-cohort, migrant status was grouped into (i) quota refugees (refugees arrived via organised resettlement), (ii) former asylum seekers (refugees arrived independently), (iii) family-reunified immigrants (arrived to join close relatives already legally residing in Denmark) and (iv) others. The majority of the “others” category came from the first cohort, which means they were our target population, but the exact category could not be determined.

Information on cause of death was retrieved from the Register of Causes of Death, coded according to the International Classification of Diseases, 10th revision (ICD-10) [27]. To obtain statistically meaningful estimates, this study only analysed specific causes with more than 10 death cases recorded among immigrant children and youths.

### Statistical analyses

Incidence rate (IR) was calculated by dividing the number of deaths by person-years at risk. IR was used to describe mortality by sex and age in both native and immigrant populations, and additionally by region of origin, migrant status, age at entry, and length of stay in immigrant children and youths. Cox modelling and hazard ratio (HR) with 95% confidence interval (95% CI) were used to compare mortality risk between natives and all immigrants, as well as between natives and immigrant groups divided by region of origin and migrant status. Given reported sex differences in immigrant mortality [15, 28, 29], Cox regression analyses differentiated by sex were conducted as well. Age was used as the timescale in the Cox models to minimize the influence of the imbalanced age distribution and delayed entry in this study. The Schoenfeld residuals tests show that the proportional hazards assumption was not violated in our models. As sensitivity analyses, we calculated the unadjusted incidence rate ratio (IRR) of all-cause mortality of native Danes versus (i) immigrants from different regions of origin and (ii) immigrants with different migrant statuses. Respective IRRs and HRs were then compared. All analyses were performed with R (version 4.4.1).

### Ethical considerations

The research adhered to the principles outlined in the Declaration of Helsinki to ensure the rights, safety, and well-being of participants were protected throughout the study. According to Danish law, studies based solely on secondary register data do not require informed consent from participants or approval from research ethics committees. Data access was granted by Statistics Denmark, and all analyses were conducted within their remote access server. All data were pseudonymized before access.

## Results

Table 1 shows the descriptive characteristics of the study cohort. The percentage of male participants (48.6%) was comparable to female participants (51.4%). Central Europe, Eastern Europe, and Central Asia (39.0%) and North Africa and Middle East (32.5%) were the regions of origin for most of the immigrant children and youths. Almost half of the participants arrived in their young adulthood (41.8%). Immigrant children and youths mainly entered as former asylum seekers (48.7%) or family-reunified immigrants (38.7%). Age at entry and mean follow-up time varied substantially. We identified 156 deaths among immigrant children and youths and 815 deaths among native children and youths. Accidents were the most common cause of death for both native (74.2%) and immigrant (51.9%) children and youths.

**Table 1:** Characteristics of the study population, stratified by sex and nativity

	Female		Male		Total *n* (column %)
	Natives n (column %)	Immigrants *n* (column %)	Natives *n* (column %)	Immigrants n (column %)	
Total *n* (row %)	242,770 (43.7)	42,757 (7.7)	230,474 (41.5)	39,704 (7.1)	555,705 (100)
Region of origin					
Central Europe, Eastern Europe, and Central Asia	-	16,644 (38.9)	-	15,525 (39.1)	32,169 (39.0)
North Africa and Middle East	-	12,553 (29.4)	-	14,255 (35.9)	26,808 (32.5)
Sub-Saharan Africa	-	5,343 (12.5)	-	5,380 (13.6)	10,723 (13.0)
Other regions	-	8,217 (19.2)	-	4,544 (11.4)	12,761 (15.5)
Age at entry					
0–4	37,948 (15.6)	5,283 (12.4)	39,265 (17.0)	5,887 (14.8)	88,383 (15.9)
5–9	31,531 (13.0)	5,660 (13.2)	34,243 (14.9)	6,385 (16.1)	77,819 (14.0)
10–14	21,689 (8.9)	4,081 (9.5)	25,607 (11.1)	4,763 (12.0)	56,140 (10.1)
15–19	45,640 (18.8)	7,989 (18.7)	39,986 (17.3)	7,364 (18.5)	100,979 (18.2)
20–24	105,962 (43.7)	19,744 (46.2)	91,373 (39.7)	15,305 (38.6)	232,384 (41.8)
Mean age at entry (year ± SD)	15.8 ± 8.0	16.4 ± 7.6	15.0 ± 8.1	15.2 ± 7.8	15.5 ± 8.0
Migrant status^a^					
Quota refugees	-	2,022 (8.1)	-	2,240 (10.1)	4,262 (9.0)
Former asylum seekers	-	9,994 (40.1)	-	12,953 (58.3)	22,947 (48.7)
Family-reunified immigrants	-	12,109 (48.5)	-	6,148 (27.7)	18,257 (38.7)
Others	-	827 (3.3)	-	858 (3.9)	1,685 (3.6)
Length of stay (year)					
0–4	-	26,956 (63.0)	-	22,595 (56.9)	49,551 (60.1)
5–9	-	7,467 (17.5)	-	7,454 (18.8)	14,921 (18.1)
10–14	-	2,896 (6.8)	-	3,344 (8.4)	6,240 (7.6)
** ≥ **15	-	5,438 (12.7)	-	6,311 (15.9)	11,749 (14.2)
Mean follow-up time (year ± SD)	6.7 ± 7.1	5.5 ± 6.4	7.2 ± 7.3	6.3 ± 6.4	6.8 ± 7.1
Status at the end of follow-up					
Death	264 (0.1)	48 (0.1)	551 (0.2)	108 (0.3)	971 (0.2)
Emigration	9,443 (3.9)	4,451 (10.4)	7,189 (3.1)	3,567 (9.0)	24,650 (4.4)
25th birthday	170,421 (70.2)	26,678 (62.4)	153,960 (66.8)	23,239 (58.5)	374,295 (67.4)
Study end	62,642 (25.8)	11,580 (27.1)	68,774 (29.9)	12,790 (32.2)	155,789 (28.0)
Cause-specific deaths^b^					
Neurological disorder	22 (8.3)	5 (10.4)	32 (5.8)	9 (8.3)	68 (7.0)
Suicide	23 (8.7)	6 (12.5)	84 (15.2)	12 (11.1)	125 (12.9)
Cancer	55 (20.8)	7 (14.6)	61 (11.1)	12 (11.1)	135 (13.9)
Accidents	83 (31.4)	8 (16.7)	236 (42.8)	38 (35.2)	365 (37.6)

^a^Only available for the sub-cohort

^b^Denominator of the percentage was the total number of deaths in the corresponding group. Only specific causes with more than 10 cases among immigrant children and youths were listed; therefore, the sum of the percentages is smaller than 100

Table 2 presents IRs of all-cause mortality. Overall, the all-cause mortality rate was higher among immigrants (IR = 3.20 per 10,000 person-years) compared to natives (IR = 2.49 per 10,000 person-years). Higher IRs were also observed across most immigrant subgroups, with the exceptions of (i) family-reunified immigrants, (ii) those arriving at ages 20–24 and (iii) those residing ≥ 15 years. Mortality rate in immigrant children and youths decreased substantially with longer duration of stay, from an initial IR of 9.39 per 10,000 person-years in the first 5 years to an IR of 0.66 per 10,000 person-years after 15 years or more of residence.

**Table 2:** Unadjusted incidence rates (IRs) of all-cause mortality of children and youths per 10,000 person-years in immigrants versus natives

	Natives			Immigrants		
	Person-years	Deaths	IR (95% CI)	Person-years	Deaths	IR (95% CI)
Total immigrants versus natives (ref)	3,268,245	815	2.49 (2.33**–**2.67)	487,708	156	3.20 (2.72**–**3.74)
Sex						
Female	1,618,501	264	1.63 (1.44**–**1.84)	235,806	48	2.04 (1.50**–**2.70)
Male	1,649,754	551	3.34 (3.07**–**3.63)	251,902	108	4.29 (3.52**–**5.18)
Region of origin						
Denmark	3,268,245	815	2.49 (2.33**–**2.67)	-	-	-
Central Europe, Eastern Europe, and Central Asia	-	-	-	179,425	50	2.79 (2.07**–**3.67)
North Africa and Middle East	-	-	-	192,897	61	3.16 (2.42**–**4.06)
Sub-Saharan Africa	-	-	-	76,837	33	4.29 (2.96**–**6.03)
Other regions	-	-	-	38,549	12	3.11 (1.61**–**5.43)
Migrant Status^a^						
Natives	1,800,028	374	2.08 (1.87**–**2.30)	-	-	-
Quota refugees	-	-	-	43,217	20	4.63 (2.83**–**7.15)
Former asylum seekers	-	-	-	154,530	53	3.43 (2.57**–**4.49)
Family-reunified immigrants	-	-	-	86,700	11	1.27 (0.63**–**2.27)
Others	-	-	-	15,944	0	-
Age at entry						
0–4	1,223,836	216	1.76 (1.54**–**2.02)	152,416	36	2.36 (1.65**–**3.27)
5–9	800,120	196	2.45 (2.12**–**2.82)	131,859	33	2.50 (1.72**–**3.51)
10–14	402,959	139	3.45 (2.90**–**4.07)	64,602	36	5.57 (3.90**–**7.71)
15–19	477,609	176	3.69 (3.16**–**4.27)	77,394	43	5.56 (4.02**–**7.48)
20–24	363,721	88	2.42 (1.94**–**2.98)	61,437	8	1.30 (0.56**–**2.57)
Length of stay						
0–4	-	-	-	75,586	71	9.39 (7.34**–**11.85)
5–9	-	-	-	106,258	45	4.23 (3.09**–**5.67)
10–14	-	-	-	77,433	25	3.23 (2.09**–**4.77)
** ≥ **15	-	-	-	228,431	15	0.66 (0.37**–**1.08)

^a^Only available for the sub-cohort

Table 3 lists HRs of all-cause mortality. Overall, immigrant children and youths had significantly higher mortality risk compared with natives (HR 1.27, 95% CI 1.07–1.51). In subgroups, immigrant children and youths from Sub-Saharan Africa had the highest risk (HR 1.77, 95% CI 1.25–2.51), driven by male immigrant children and youths (HR 1.89, 95% CI 1.27–2.83). Further, quota refugees (Females: HR 2.52, 95% CI 1.18–5.40; Males: HR 2.29, 95% CI 1.31–4.00) and former asylum seekers (Females: HR 1.58, 95% CI 0.93–2.70; Males: HR 1.54, 95% CI 1.10–2.17) had higher risks in both sexes.

**Table 3:** Hazard ratios (HRs) of sex-specific all-cause mortality for immigrant children and youths compared with natives, grouped by region of origin and migrant status, adjusted for age

	Female immigrants		Male immigrants		Total immigrants	
	HR	95% CI	HR	95% CI	HR	95% CI
Region of origin						
Central Europe, Eastern Europe, and Central Asia	0.97	0.57–1.67	1.20	0.85–1.67	1.13	0.85–1.50
North Africa and Middle East	1.51	0.98–2.34	1.09	0.79–1.51	1.25	0.96–1.62
Sub-Saharan Africa	1.39	0.69–2.81	1.89	1.27–2.83**	1.77	1.25–2.51**
Other regions	0.97	0.36–2.62	1.38	0.69–2.78	1.09	0.61–1.93
Total	1.24	0.91–1.69	1.27	1.04–1.56*	1.27	1.07–1.51**
Migrant status^a^						
Quota refugees	2.52	1.18–5.40*	2.29	1.31–4.00**	2.40	1.53–3.77***
Former asylum seekers	1.58	0.93–2.70	1.54	1.10–2.17*	1.63	1.23–2.19***
Family-reunified immigrants	0.73	0.32–1.66	0.50	0.21–1.21	0.55	0.30–1.01
Others	-^b^	-	-	-	-	-
Total	1.30	0.86–1.95	1.32	0.99–1.76	1.32	1.04–1.67*

*: *p* < 0.05 **: *p* < 0.01 ***: *p* < 0.001

^a^Only available for the sub-cohort

^b^Not applicable due to few cases

For cause-specific mortality analyses, deaths from cancer (C00–D09), neurological disorder (G00–G99), accidents (V00–X58) and suicide (X71–X83) accounted for 10 or more total cases. However, differences in cancer and accident deaths between immigrant and native children and youths were not pronounced (Table A1 and Table A2). The results on the differences in deaths from neurological disorders and suicide are shown in Tables 4 and 5. Both quota refugees (HR 6.01, 95% CI 2.10–17.24) and former asylum seekers (HR 2.58, 95% CI 1.07–6.25) had higher neurological disorder mortality risk compared to natives; female quota refugees had the highest risk (HR 13.2, 95% CI 3.59–48.36). Further, female immigrant children and youths from North Africa and Middle East had higher suicide mortality risk compared to natives (HR 3.86, 95% CI 1.47–10.45).

**Table 4:** Hazard ratios (HRs) of sex-specific neurological disorder mortality for immigrant children and youths compared with natives, grouped by region of origin and migrant status, adjusted for age

	Female immigrants		Male immigrants		Total immigrants	
	HR	95% CI	HR	95% CI	HR	95% CI
Region of origin						
Central Europe, Eastern Europe, and Central Asia	0.82	0.11–6.10	2.22	0.78–6.27	1.66	0.66–4.14
North Africa and Middle East	2.49	0.74–8.32	0.99	0.24–4.13	1.57	0.63–3.93
Sub–Saharan Africa	2.05	0.28–15.19	2.53	0.61–10.56	2.37	0.74–7.57
Other regions	–^a^	–	3.31	0.45–24.29	1.57	0.22–11.40
Total	1.55	0.59–4.10	1.83	0.87–3.83	1.73	0.96–3.11
Migrant status^b^						
Quota refugees	13.20	3.59–48.36***	2.26	0.30–17.05	6.01	2.10–17.24***
Former asylum seekers	2.67	0.58–12.18	2.42	0.81–7.19	2.58	1.07–6.25*
Family-reunified immigrants	-	-	1.49	0.20–11.22	0.79	0.11–5.82
Others	-	-	-	-	-	-
Total	3.12	1.07–9.14*	2.05	0.81–5.20	2.44	1.21–4.93*

*: *p* < 0.05 **: *p* < 0.01 ***: *p* < 0.001

^a^Not applicable due to few cases

^b^Only available for the sub-cohort

**Table 5:** Hazard ratios (HRs) of sex-specific suicide mortality for immigrant children and youths compared with natives, grouped by region of origin and migrant status, adjusted for age

	Female immigrants		Male immigrants		Total immigrants	
	HR	95% CI	HR	95% CI	HR	95% CI
Region of origin						
Central Europe, Eastern Europe, and Central Asia	0.81	0.11**–**6.02	-^a^	-	0.17	0.02**–**1.24
North Africa and Middle East	3.86	1.47**–**10.45**	0.89	0.36**–**2.19	1.51	0.79**–**2.90
Sub-Saharan Africa	-	-	2.04	0.75**–**5.55	1.69	0.62**–**4.58
Other regions	-	-	3.07	0.97**–**9.73	1.74	0.55**–**5.49
Total	1.72	0.70**–**4.22	0.91	0.50**–**1.68	1.09	0.66**–**1.80
Migrant status^b^						
Quota refugees	-	-	2.11	0.51**–**8.73	1.72	0.42**–**7.04
Former asylum seekers	3.12	0.90**–**10.88	1.13	0.45**–**2.84	1.62	0.77**–**3.40
Family-reunified immigrants	0.91	0.12**–**6.97	-	-	0.29	0.04**–**2.11
Others	-	-	-	-	-	-
Total	1.65	0.54**–**5.01	0.92	0.41**–**2.04	1.10	0.58**–**2.10

*: *p* < 0.05 **: *p* < 0.01 ***: *p* < 0.001

^a^Not applicable due to few cases

^b^Only available for the sub-cohort

Unadjusted IRRs regarding all-cause mortality were consistent with the main findings obtained via Cox models (Table A3).

## Discussion

In the study cohort of children and youths under 25, immigrant individuals experienced higher all-cause mortality risk compared with native-born Danes. These results are consistent with those reported in register-based studies in other European countries. The excess was most pronounced among immigrants originating from Sub-Saharan Africa and refugees. Cause-specific analyses identified elevated neurological disorder and suicide mortality in some immigrant subgroups. Mortality rate among immigrant children and youths declined with longer length of stay.

### All-cause mortality

Immigrant children and youths in the study had a higher overall risk of dying compared to native-born individuals. This matched a previously observed U-shaped pattern in migrant mortality, where risk of death is highest among the youngest and oldest migrants and lowest for those who migrated at intermediate ages [30]. One possible explanation could be that the “healthy immigrant effect” seen in adults—where healthier or more capable people are more likely to migrate—is much weaker in children. Unlike adults, children migrate because their guardians decide they will, not because they choose to themselves. As a result, the health-related advantages often seen in adult migrants are less likely to apply to children [14, 30]. Another possible explanation is cultural influences: those who migrate at a young age are more likely to adopt the norms and behaviours of the host country, so any protective health advantage associated with the original culture (e.g. lower rates of smoking, reduced alcohol consumption, and blander dietary patterns) may be less significant for child migrants [30]. Hence, the drop in mortality rate among those ages 20–24 may reflect self-selection of adult immigrants and established health habits before migration. Consanguinity, socioeconomic factors, and inadequate care have been argued to contribute to higher infant mortality in the migrant population in Denmark [31] and could partly explain the excess risk in our study population.

Our study further suggested that all-cause mortality was influenced by migrant status and region of origin, with refugee status and Sub-Saharan Africa origin contributing to higher risks. Significantly higher all-cause mortality in refugee children and youths may be explained by excess pre- and peri-migration risk factors, such as exposure to conflict, undernutrition and trauma before their departure, as well as stress, overcrowding and exhaustion during their migration journey. Further, the Danish refugee selection criteria up to 2005 included consideration of medical need when selecting quota refugees and approving humanitarian residence permits [32, 33], which could have increased the proportion of sick individuals within the refugee population. Additional post-migration risk factors – e.g. uncertainty and restrictions during stays in asylum centres and the absence of supportive integration programmes, which are usually offered to quota refugees and family-reunified immigrants [34] – may increase mortality risk in former asylum seekers [35].

Our finding of elevated overall mortality risk among children and youths from Sub-Saharan Africa aligns with the previous study of adult immigrants [15]. Poorer health outcomes in this group may be associated with structural socioeconomic status (SES) disadvantages [16]. In our study population, this excess risk was only statistically significant in males; in the adult population, however, it was more pronounced in females [15]. This different sex pattern across age groups may reflect factors specific to adulthood, including pregnancy-related mortality among female African migrants [36, 37].

Finally, we found that the all-cause mortality rate decreased with length of stay. The same pattern was discovered in Sweden but, owing to the small sample size of the under-18 age group [38], lacked statistical significance. This result contrasts with the idea of “unhealthy assimilation”, i.e. that adult migrants adopt unhealthy habits over time and their health becomes similar to that of the native population [17, 38, 39]. Our findings instead suggest that the health disadvantages of immigrant children may diminish with time and the supposed stability and integration conferred by longer stays.

### Cause-specific mortality

Despite the limited number of events, we observed some indication of excess mortality due to neurological disorders among children and youths arriving as quota refugees or asylum seekers. Previous research has reported higher risks of neurological and developmental conditions in children of immigrant families, potentially linked to prenatal [40–42], genetic [40, 41], and peri-migration [42] factors. Additionally, we found higher suicide mortality among female immigrant children and youths from North Africa and Middle East, aligning with earlier evidence of elevated suicidal behavior in female migrants [43, 44]. Psychosocial stressors, such as challenges in social integration and transition to adulthood [45], together with potential barriers to access and utilize mental healthcare [46, 47], may play a role, particularly among girls.

### Strengths and limitations

This is the first national register–based cohort study focused on the mortality differences between immigrant and native children and youths in Denmark. Use of the nationwide high-quality register data of Denmark [48] enabled us to build a large study cohort with a long time span. Also, the reliable data linkage via personal identification number was not subject to self-reported bias or non-response.

Nevertheless, the study had several limitations. First, despite the large sample size, the outcome (i.e. death) itself was rare, which led to wide confidence intervals and limited further stratification, such as cross-classification. This problem was particularly evident in cause-specific mortality, where the few death cases caused unstable statistical outcomes and masked potential mortality risks. Additionally, when building the Cox model, adjustment for SES (e.g. education, income) was lacking due to the difficulties in measuring SES in young populations. Considering the structural SES disadvantages faced by immigrants discussed earlier, the absence of adjustment for these well-established confounders may suggest overestimation of observed associations. The potential for residual confounding should be considered when interpreting the findings. Bias could also come from the presence of systematic missingness in several demographic covariates. As a result, analyses restricted to complete cases may not be fully representative of the target population. Besides, construction of the study cohort was based on the personal identification numbers of participants, which meant that only immigrant children and youths who had already obtained their residence permits were included. Therefore, the results of this study cannot be generalized to those who are waiting in asylum centres. What’s more, the study period was inconsistent, as immigrants entering between 2016 and 2019 were not included due to data availability. This may limit representativeness, since migration patterns and immigrant characteristics could differ across calendar years.

## Conclusion

This study demonstrates that immigrant children and youths in Denmark face higher mortality risks compared with their native peers, with particularly elevated risks among refugees and those originating from Sub-Saharan Africa. However, their health disadvantages diminished with longer stay. These findings underscore the need for targeted health and integration policies to address the vulnerabilities of immigrant children and youths. They also highlight the potential long-term benefits of a supportive environment.

## Supplementary Information

Below is the link to the electronic supplementary material.

ESM 1 Supplementary Material 1 (DOCX 29.2 KB)

## Data Availability

The original data was not collected by the authors, but made available by record-linkage, using the Danish unique individual civil registration number (CPR) and the Danish administrative registers. According to a permission received from the Danish Data Protection Agency (DDPA), the principal investigator (MN) is not allowed to forward the record-linkage dataset to other researchers outside the project group. The corresponding authors can provide metadata (tables) on request to other researchers, for example showing results of alternative analytic strategies.

## Abbreviations

HR: Hazard Ratio
ICD-10: International Classification of Diseases, 10th revision
IR: Incidence Rate
IRR: Incidence Rate Ratio
MMA: Migrant Mortality Advantage
SES: Socioeconomic Status
WHO: World Health Organization
95% CI: 95% Confidence Interval
