# Supplementary material for: Mortality differences between immigrant and native children and youths in Denmark: a nationwide cohort study focusing on refugees and family-reunified immigrants
Source: Eur J Pediatr. 2026 Jun 13;185(7):496. doi: 10.1007/s00431-026-07159-z (PMC13264540; doi:10.1007/s00431-026-07159-z)
Supplement: Supplementary file 1 — Supplementary Material 1 (DOCX 29.2 KB) [file 431_2026_7159_MOESM1_ESM.docx]

**Appendix tables**

**Table A1.** Hazard ratios (HRs) of sex-specific cancer mortality for immigrant children and youths compared with natives, grouped by region of origin and migrant status, adjusted for age.

|  | Female immigrants | | Male immigrants | | Total immigrants | |
| --- | --- | --- | --- | --- | --- | --- |
|  | HR | 95% CI | HR | 95% CI | HR | 95 % CI |
| Region of origin |  |  |  |  |  |  |
| Central Europe, Eastern Europe, and Central Asia | 0.99 | 0.31**–**3.15 | 1.77 | 0.77**–**4.11 | 1.41 | 0.71**–**2.77 |
| North Africa and Middle East | 0.99 | 0.31**–**3.16 | 0.78 | 0.24**–**2.48 | 0.87 | 0.38**–**1.99 |
| Sub-Saharan Africa | -^a^ | - | 1.99 | 0.62**–**6.33 | 1.10 | 0.35**–**3.47 |
| Other regions | 1.24 | 0.17**–**9.03 | - | - | 0.71 | 0.10**–**5.11 |
| Total | 0.86 | 0.39**–**1.90 | 1.29 | 0.69**–**2.39 | 1.09 | 0.67**–**1.78 |
| Migrant status^b^ |  |  |  |  |  |  |
| Quota refugees | - | - | 1.40 | 0.19**–**10.31 | 0.69 | 0.10**–**4.98 |
| Former asylum seekers | 1.26 | 0.39**–**4.13 | 1.36 | 0.48**–**3.87 | 1.32 | 0.60**–**2.88 |
| Family-reunified immigrants | - | - | 0.84 | 0.11**–**6.16 | 0.32 | 0.04**–**2.34 |
| Others | - | - | - | - | - | - |
| Total | 0.57 | 0.17**–**1.85 | 1.18 | 0.49**–**2.84 | 0.87 | 0.43**–**1.74 |

*: p<0.05 **: p<0.01 ***: p<0.001

a: Not applicable due to few cases

b: Only available for the sub-cohort

**Table A2.** Hazard ratios (HRs) of sex-specific accident mortality for immigrant children and youths compared with natives, grouped by region of origin and migrant status, adjusted for age.

|  | Female immigrants | | Male immigrants | | Total immigrants | |
| --- | --- | --- | --- | --- | --- | --- |
|  | HR | 95% CI | HR | 95% CI | HR | 95 % CI |
| Region of origin |  |  |  |  |  |  |
| Central Europe, Eastern Europe, and Central Asia | 1.08 | 0.44**–**2.66 | 1.01 | 0.58**–**1.76 | 1.03 | 0.64**–**1.65 |
| North Africa and Middle East | 0.44 | 0.11**–**1.77 | 0.97 | 0.58**–**1.64 | 0.88 | 0.54**–**1.44 |
| Sub-Saharan Africa | 0.55 | 0.08**–**3.99 | 1.24 | 0.59**–**2.64 | 1.11 | 0.55**–**2.23 |
| Other regions | - | - | 1.19 | 0.38**–**3.73 | 0.70 | 0.22**–**2.18 |
| Total | 0.66 | 0.32**–**1.36 | 1.04 | 0.74**–**1.47 | 0.95 | 0.70**–**1.30 |
| Migrant status^a^ |  |  |  |  |  |  |
| Quota refugees | 3.41 | 1.05**–**11.06* | 0.89 | 0.22**–**3.60 | 1.64 | 0.67**–**4.00 |
| Former asylum seekers | -^b^ | - | 1.28 | 0.72**–**2.28 | 1.07 | 0.61**–**1.90 |
| Family-reunified immigrants | - | - | 0.72 | 0.23**–**2.28 | 0.39 | 0.13**–**1.23 |
| Others | - | - | - | - | - | - |
| Total | 0.45 | 0.14**–**1.45 | 1.03 | 0.62**–**1.70 | 0.87 | 0.55**–**1.38 |

*: p<0.05 **: p<0.01 ***: p<0.001

a: Only available for the sub-cohort

b: Not applicable due to few cases

**Table A3.** Comparison between hazard ratios (HRs) and incidence rate ratios (IRRs) of all-cause mortality in total immigrant children and youths.

|  | Total immigrants | | | |
| --- | --- | --- | --- | --- |
|  | HR | 95% CI | IRR | 95 % CI |
| Region of origin |  |  |  |  |
| Central Europe, Eastern Europe, and Central Asia | 1.13 | 0.85**–**1.50 | 1.12 | 0.83**–**1.47 |
| North Africa and Middle East | 1.25 | 0.96**–**1.62 | 1.27 | 0.97**–**1.63 |
| Sub-Saharan Africa | **1.77** | **1.25–2.51^a^** | **1.73** | **1.20–2.41** |
| Other regions | 1.09 | 0.61**–**1.93 | 1.27 | 0.67**–**2.13 |
| Total | **1.27** | **1.07–1.51** | **1.28** | **1.08–1.52** |
| Migrant status^b^ |  |  |  |  |
| Quota refugees | **2.40** | **1.53–3.77** | **2.24** | **1.38–3.42** |
| Former asylum seekers | **1.63** | **1.23–2.19** | **1.66** | **1.23–2.20** |
| Family-reunified immigrants | 0.55 | 0.30**–**1.01 | 0.62 | 0.32**–**1.07 |
| Others | -^c^ | - | - | - |
| Total | **1.32** | **1.04–1.67** | **1.35** | **1.06–1.70** |

a: Values in bold are statistically significant (p value < 0.05)

b: Only available for the sub-cohort

c: Not applicable due to few cases
